# Atorvastatin Promotes Phagocytosis and Attenuates Pro-Inflammatory Response in Human Retinal Pigment Epithelial Cells

**DOI:** 10.1038/s41598-017-02407-7

**Published:** 2017-05-24

**Authors:** Bo Tian, Ahmad Al-Moujahed, Peggy Bouzika, Yijun Hu, Shoji Notomi, Pavlina Tsoka, Joan W. Miller, Haijiang Lin, Demetrios G. Vavvas

**Affiliations:** 1 Retina Service, Angiogenesis Laboratory, Department of Ophthalmology, Massachusetts Eye and Ear Infirmary, Harvard Medical School, Boston, MA 02114 United States; 2 Department of Pathology, Boston University School of Medicine, Boston, MA 02118 United States; 3 State Key Laboratory of Ophthalmology, Zhongshan Ophthalmic Center, Sun Yat-sen University, Guangzhou, 510060 China; 4 Department of Ophthalmology & Visual Sciences, University of Massachusetts Medical School, Worcester, MA 01605 United States

## Abstract

Phagocytosis of daily shed photoreceptor outer segments is an important function of the retinal pigment epithelium (RPE) and it is essential for retinal homeostasis. RPE dysfunction, especially impairment of its phagocytic ability, plays an essential role in the pathogenesis of age-related macular degeneration (AMD). Statins, or HMG CoA (3-hydroxy-3-methylglutaryl-coenzyme A) reductase inhibitors, are drugs with multiple properties that have been extensively used to treat hyperlipidemia. However, their effect on RPE cells has not been fully elucidated. Here we report that high dose atorvastatin increased the phagocytic function of ARPE-19 cells, as well as rescue the cells from the phagocytic dysfunction induced by cholesterol crystals and oxidized low-density lipoproteins (ox-LDL), potentially by increasing the cellular membrane fluidity. Similar effects were observed when evaluating two other hydrophobic statins, lovastatin and simvastatin. Furthermore, atorvastatin was able to block the induction of interleukins IL-6 and IL-8 triggered by pathologic stimuli relevant to AMD, such as cholesterol crystals and ox-LDL. Our study shows that statins, a well-tolerated class of drugs with rare serious adverse effects, help preserve the phagocytic function of the RPE while also exhibiting anti-inflammatory properties. Both characteristics make statins a potential effective medication for the prevention and treatment of AMD.

## Introduction

AMD accounts for 8.7% of all blindness worldwide and is the most common cause of blindness in developed countries, especially in people older than 60 years^1^. Eighty-five percent of patients present with the dry (atrophic) form of the disease, while the wet (neo-vascular) form affects about 15% of individuals, and usually develops on a background of dry AMD. While effective treatments are available for wet AMD^2, 3^, there is rarely successful treatment for dry AMD^4^.

The retinal pigment epithelium (RPE) is a monolayer of specialized pigmented epithelial cells that lies between the neural retina and the choroids^5^. The structural and functional integrity of the RPE is fundamental for maintaining the function of the neuroretina^5^. The photoreceptor outer segments (POSs), responsible for converting light to electric impulses, renew themselves by shedding packets of distal outer segment tips once daily. Shed POSs are removed and recycled through RPE phagocytosis. RPE dysfunction, and in particular impairment of its phagocytic ability, has an essential role in the pathogenesis of age-related macular degeneration AMD^5–9^.

Numerous studies have documented that lipids as well as lipid oxidation products negatively modulate RPE function^10^. Indeed, the age-related accumulation of lipids resulting from photoreceptor turnover or the internalization of low-density lipoproteins (LDL) is a burden on RPE cells^11, 12^. Moreover, accumulation of oxidized lipids and lipoproteins has been found in Bruch’s membrane and is thought to be an early event in development of AMD^13, 14^. In addition, oxidized low density lipoproteins (ox-LDL) are internalized by the RPE and interfere with the photoreceptor turnover and proper lysosomal function^15^, thus suggesting a feed-forward loop which contributes to the pathogenesis of AMD.

In addition to lipids, inflammation is another significant factor in AMD pathogenesis^16^. Specifically, the pro-inflammatory cytokines interleukin-6 (IL-6) and interleukin-8 (IL-8) as well as NLRP3 inflammasome are associated with AMD development and/or progression^17–23^. We have also previously shown that cholesterol crystals, an insoluble unesterified form of serum cholesterol, induce the secretion of IL-6 and IL-8, and the expression of pro-IL-1β in the human retinal pigment epithelium cell line ARPE-19^24^.

Statins, or HMG CoA (3-hydroxy-3-methylglutaryl-coenzyme A) reductase inhibitors, which block cholesterol biosynthesis via the mevalonate pathway and upregulate LDL receptor expression, have been extensively used to lower serum cholesterol levels. Since cardiovascular risk factors are also associated with AMD, interventions that reduce cardiovascular risk factors, such as statins, may be useful in AMD. Multiple studies^25–29^ have investigated the relationship between lipid status, statin use, and the development and progression of AMD without reaching a conclusion about whether statins can be beneficial in the treatment or prevention of AMD. Recently, our exploratory phase I/II clinical study showed that high-dose atorvastatin may result in resolution of drusenoid pigment epithelial detachments (PEDs) and improvement in visual acuity in a high-risk subgroup of AMD patients^30^. However, the exact mechanism by which statins exert their therapeutic effect is not completely understood.

Currently, there are many statins used to treat hyperlipidemia, and although they share similarities, differences in their water solubility and potency exist^31–33^. Apart from their lipid-lowering action, statins also have multiple pleiotropic effects, including inhibition of inflammatory responses, improvement of endothelial function, and stabilization of atherosclerotic plaques^34^. Additionally, statins have been found to enhance the phagocytic function of human peripheral blood cells *in vitro* ^35^. However, none of these effects have been studied in RPE cells.

In this study, we wanted to investigate the effect of statins, cholesterol crystals and ox-LDL on the phagocytic function of a human RPE cell line, ARPE-19, which possesses similar phagocytic machinery as RPE *in situ* ^36^. In addition, we wished to explore whether ox-LDL is able to induce pro-inflammatory mediators in ARPE-19, similar to cholesterol crystals^24^, and if so, whether this response can be down-regulated by statins.

## Results

### Lipophilic statins increase the phagocytic function of ARPE-19 cells

**Figure 1:**
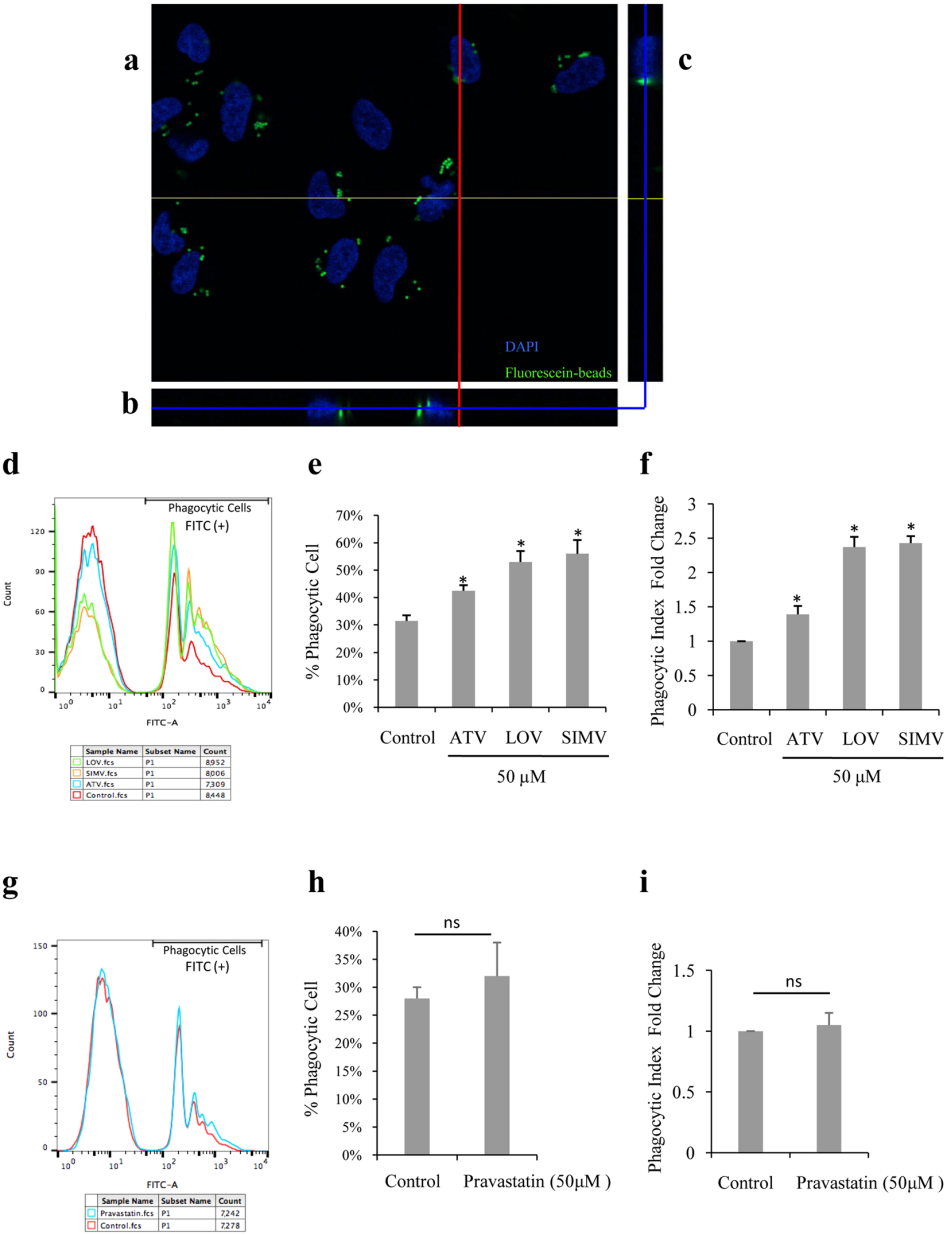
Lipophilic statins increase the phagocytic function of ARPE-19 cells. (**a**–**c**) ARPE-19 cells were incubated with polystyrene microspheres (5 × 10^7^ beads/ml) for 6 hours and then imaged by confocal microscopy collecting a stack of 50 images. (**a**) A confocal stack showing fluorescein-labeled beads (green) inside ARPE-19 cells, located proximal to DAPI-labeled nuclei (blue). The depth of image A within the stack is indicated by blue lines in (**b**) and (**c**). (**b**) A view through the same stack at the place denoted by the yellow line in (**a**). (**c**) A view through the same stack at the place denoted by the red line in (**a**). (**d**,**g**) Count of phagocytic cells (Y axis), represented by number of FITC-positive cells, and fluorescence intensity (X axis), as determined by flow cytometry of ARPE-19 cells incubated with fluorescein-labeled carboxylate microspheres and treated with different statins. **(e**,**h**) Quantification of the percentage of phagocytic cells shown in (**d**) and (**g**) separately. (**f**,**i**) Quantification of the fold change of phagocytic index (or mean fluorescence intensity). The results are expressed as mean ± SE. *p < 0.05 versus control group.

To validate a method that reliably measures the phagocytic function of the ARPE-19 cell line, cells were incubated with fluorescein-labeled polystyrene microspheres (5 × 10^7^ beads/ml) for 6 hours, and then imaged using a confocal microscopy collecting a stack of 50 images. The cells were shown to be able to actively internalize the particles (Fig. 1a–c), indicating that polystyrene microspheres can be phagocytized by ARPE-19 cells under these established conditions, and that this method can properly evaluate the phagocytic function of ARPE-19 cells.

Since statins have been reported to enhance the phagocytic function of human peripheral blood phagocytes *in vitro* ^35^, we investigated the effect of statins on the phagocytic function of human RPE cells. ARPE-19 cells were thus incubated with polystyrene microspheres along with 50 µM of either a lipophilic statin, such as atorvastatin, lovastatin and simvastatin, or a hydrophilic statin, such as pravastatin, individually. The percentage of phagocytic ARPE-19 cells was measured by flow cytometry 6 hours after treatment with different statins. We further examined the phagocytic index, measured as intensity of fluorescence of engulfed particles per cell, and any changes observed after the treatment of ARPE-19. As shown in Fig. 1d and e, atorvastatin, lovastatin, or simvastatin significantly increased the percentage of ARPE-19 phagocytic cells from 31% to 43%, 53% and 56%, respectively (*p* < 0.05). We also noted that atorvastatin, lovastatin and simvastatin promoted the phagocytic index 1.39-, 2.37-, and 2.43-fold, respectively, compared with the control group (*p* < 0.05) (Fig. 1f). However, pravastatin at the same concentration had no effect on the phagocytic function of ARPE-19 cells (Fig. 1g–i). This indicates that all three lipophilic statins tested, but not the hydrophilic statin, increase the phagocytic function of ARPE-19 cells.

### Atorvastatin increases the phagocytic function of ARPE-19 cells in a dose-dependent manner

**Figure 2:**
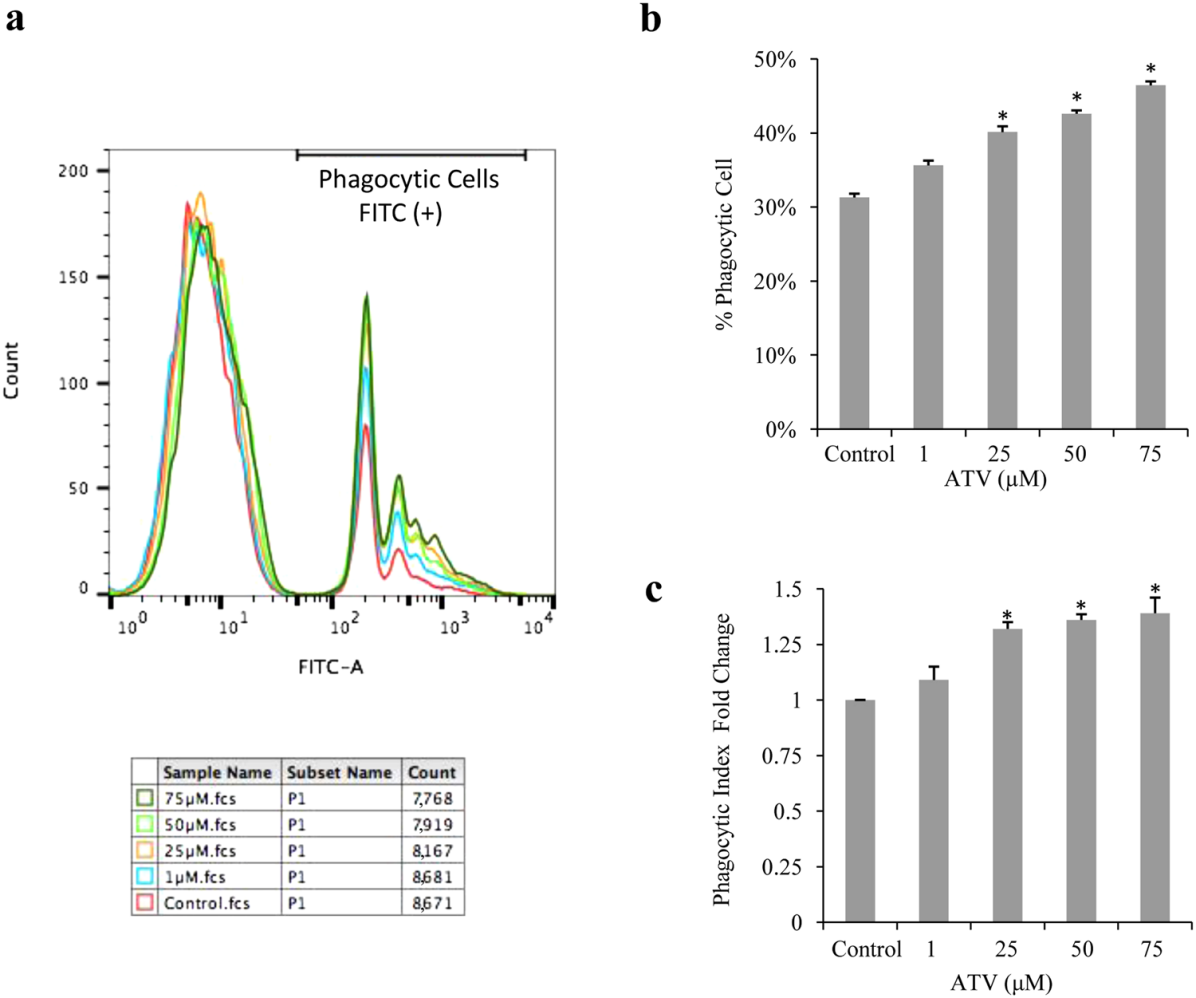
Atorvastatin increases the phagocytic function of ARPE-19 cells in a dose-dependent manner. (**a**) Count of phagocytic cells (Y axis), represented by number of FITC-positive cells, and fluorescence intensity (X axis), as determined by flow cytometry of ARPE-19 cells incubated with fluorescein-labeled carboxylate microspheres and treated with different concentrations of atorvastatin. (**b**) Quantification of the percentage of phagocytic cells shown in (**a**). (**c**) Quantification of the fold change of phagocytic index (or mean fluorescence intensity). The results are expressed as mean ± SE. *p < 0.05 versus control group.

Our recent clinical study demonstrates that high-dose atorvastatin induces the regression of drusen deposits without atrophy or neoascularization, and leads to improved visual acuity in a high-risk subgroup of AMD patients with large (>300 μm in diameter and more than 100 μm in height), soft drusenoid deposits^30^. Therefore, we further investigated the effect of different doses of atorvastatin on the phagocytic function of ARPE-19 cells. The cells were thus incubated with fluorescent polystyrene microspheres along with different doses (1, 25, 50, 75 µM) of atorvastatin for 6 hours. We found that 25, 50, or 75 µM of atorvastatin increases the percentage of phagocytic ARPE-19 cells to 40%, 42%, and 46%, respectively, compared to the baseline percentage (31%) of the control group (*p* < 0.05) (Fig. 2a and b). Moreover, the phagocytic index was significantly increased 1.32-, 1.36- and 1.39-fold after incubation with 25, 50, or 75 µM of atorvastatin, respectively (*p* < 0.05) (Fig. 2c). These data clearly indicate that atorvastatin increases the phagocytic function of ARPE-19 in a dose-dependent manner.

### Atorvastatin increases ARPE-19 cell membrane fluidity

**Figure 3:**
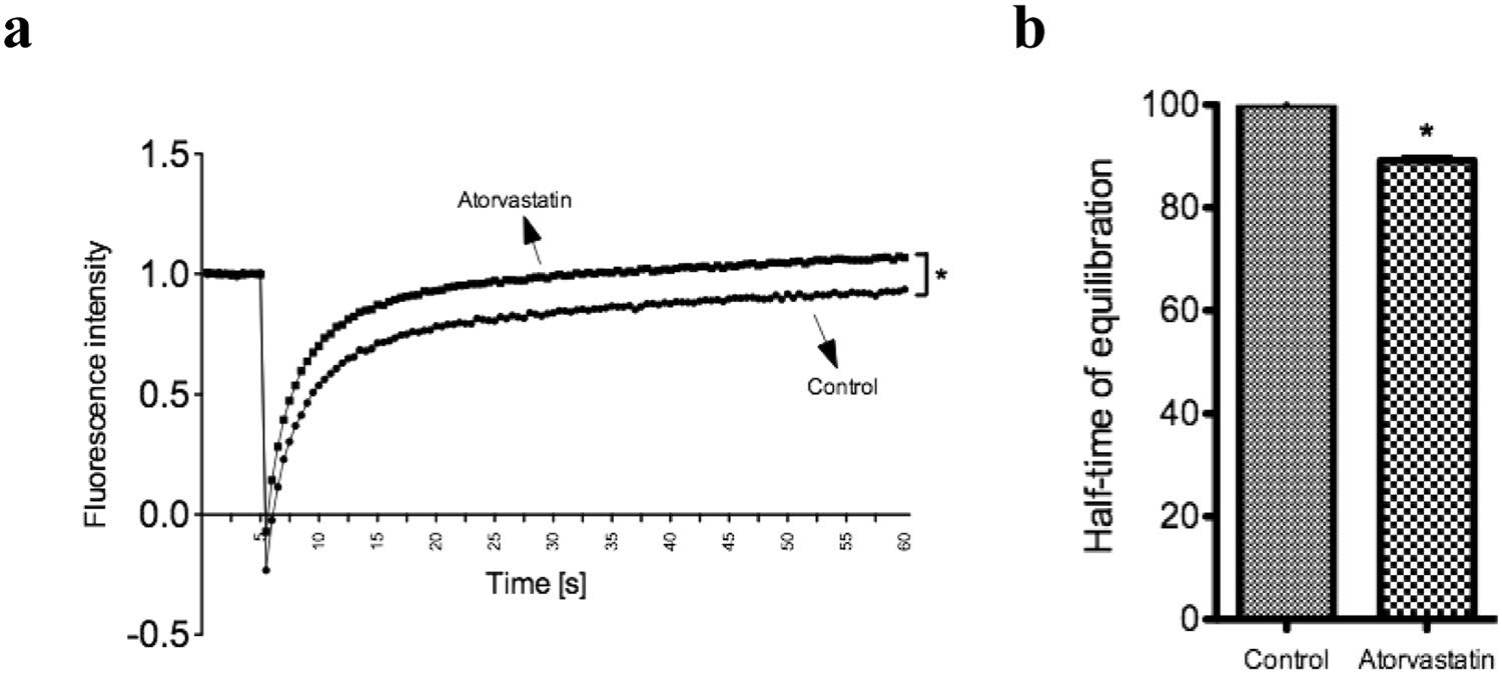
Atorvastatin increases ARPE-19 cell membrane fluidity. FRAP measurements of ARPE-19 were done 3 hours after treatment with 50 µM atorvastatin using the BODIPY® FL C12 dye. (**a**) An average recovery curve of BODIPY® FL C12 in control (n = 16 cells) or atorvastatin-treated (n = 16 cells) ARPE-19 cells. (**b**) An average of normalized half-time of equilibration of BODIPY® FL C12 in control (n = 16 cells) or atorvastatin-treated (n = 16 cells) ARPE19 cells. Data from two independent experiments. The results are expressed as mean ± SE. *p < 0.05 versus control group.

Membrane fluidity has been shown to positively modulate phagocytosis in macrophages^37, 38^. Statins have been shown to affect membrane fluidity^39^ and enhance the phagocytic activity of macrophages^35, 37, 38^. For this reason, we explored whether atorvastatin can modulate the RPE cell membrane fluidity. ARPE-19 cells were incubated with 50 µM atorvastatin for 3 hours and BODIPY® FL C12 dye for 30 minutes. FRAP measurements were made by photo-bleaching a microscopic area of the cell membrane and the recovery of fluorescence within the bleached area was assessed by repetitive scanning across the cell surface with an attenuated laser beam, as described in the methods. Our results showed that atorvastatin treatment increased the recovery of fluorescence in the membrane of ARPE-19 cells after photo-bleaching (Fig. 3a). In addition, it decreased the half-time of fluorescence equilibration compared to the control group (*p* < 0.05) (Fig. 3b), Both findings indicate that atorvastatin increases the membrane fluidity of ARPE-19 cells. This increase could, at least partially, explain the increase in phagocytic function of ARPE-19 cells after atorvastatin treatment.

### Atorvastatin protects ARPE-19 from impairment of phagocytosis induced by cholesterol crystals and ox-LDL

**Figure 4:**
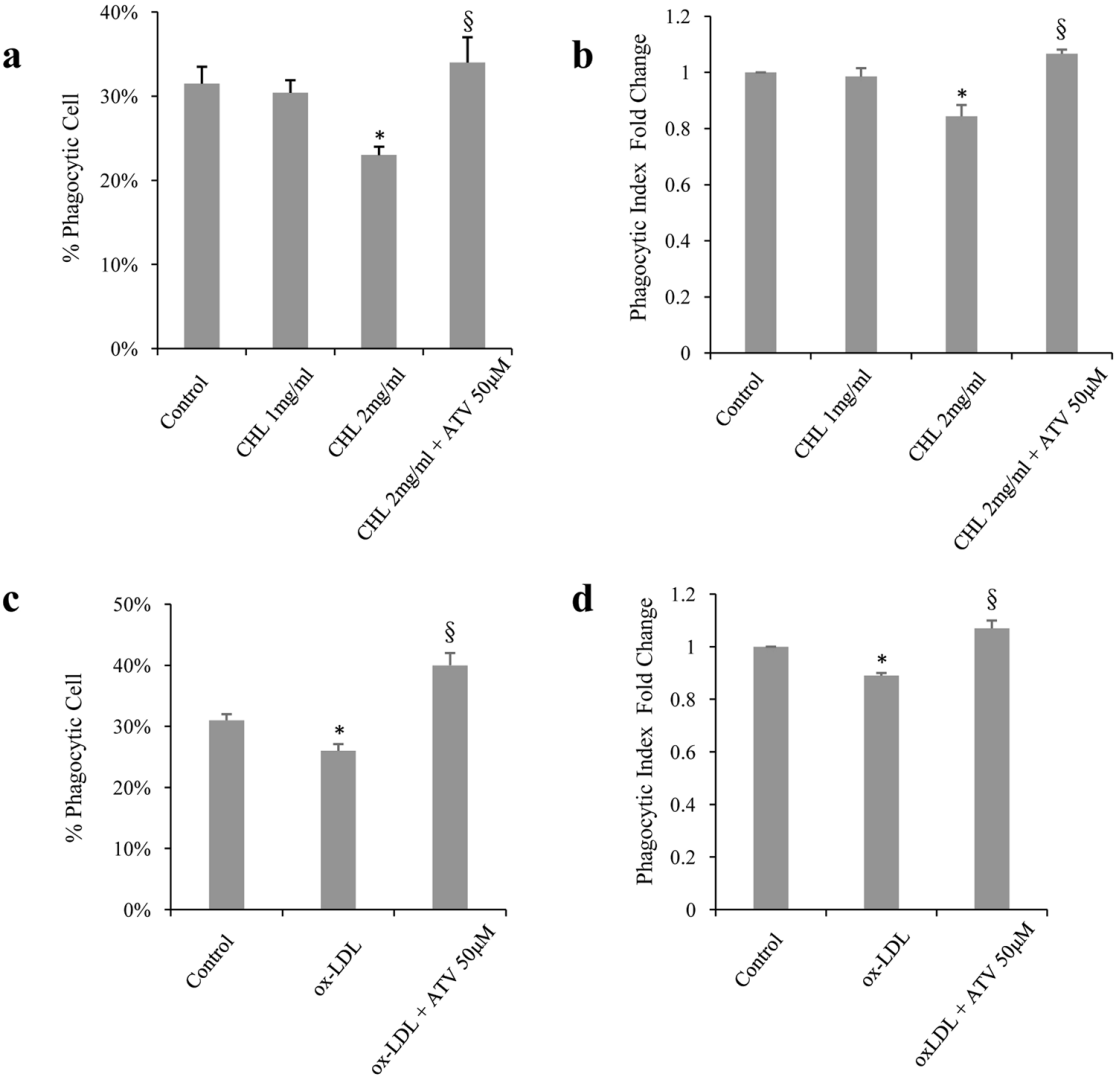
Atorvastatin protects ARPE-19 from impairment of phagocytosis induced by cholesterol crystals and ox-LDL. (**a**) Quantification of (**a**,**c**) the percent of phagocytic cells, represented by beads-positive cells, and (**b**,**d**) the fold change of phagocytic index (or mean fluorescence intensity), as determined by flow cytometry. ARPE-19 cells were incubated with fluorescein-labeled microspheres and treated with (**a**,**b**) 1 or 2 mg/ ml cholesterol crystals (CHL) for 6 hours, with or without pretreatment with 50 μM of atorvastatin (ATV) for 6 hours, or (**c**,**d**) 300 μg/ml ox-LDL for 18 hours, with or without pretreatment for 6 hours with 50 μM of atorvastatin for 6 hours. *p < 0.05 versus control group. ^§^ *p* < 0.05 versus 2 mg/ml CHL group or ox-LDL treated group. The results are expressed as mean ± SE.

Since cholesterol crystals and ox-LDL have deleterious effects on cellular functions^15, 40–42^, we sought to study their effects on the phagocytic function of RPE cells because phagocytic impairment is linked to RPE malfunction and AMD^43^. ARPE-19 cells were treated with 2 mg/ml cholesterol crystals for 6 hours, or 300 µg/ml ox-LDL for 18 hours. The phagocytic function was then assessed by flow cytometry, as described in the methods. Cholesterol crystals significantly decreased the percentage of phagocytic cells from 31% to 22% (*p* < 0.05) (Fig. 4a), and the phagocytic index of ARPE-19 cells 0.8-fold compared with the control group (*p* < 0.05) (Fig. 4b). Similarly, ox-LDL decreased the percentage of phagocytic cells from 31% to 26% (*p* < 0.05) (Fig. 4c), and the phagocytic index of cells 0.89-fold compared with the control (*p* < 0.05) (Fig. 4d).

Since atorvastatin increases the phagocytic function of ARPE-19, we checked whether it could help preserve the phagocytic properties compromised by cholesterol crystals and ox-LDL. Pretreating the cells with 50 µM atorvastatin for 6 hours completely reversed the decrease in the percentage of phagocytic cells and phagocytic index induced by cholesterol crystals (Fig. 4a and b) and ox-LDL (Fig. 4c and d).

Taken together, these results indicate that pretreatment with atorvastatin prevents the impairment of the phagocytic function of ARPE-19 cells that is induced by cholesterol crystals or ox-LDL.

### Atorvastatin inhibits IL-6 and IL-8 secretion induced by cholesterol crystals and ox-LDL in ARPE-19 cells

**Figure 5:**
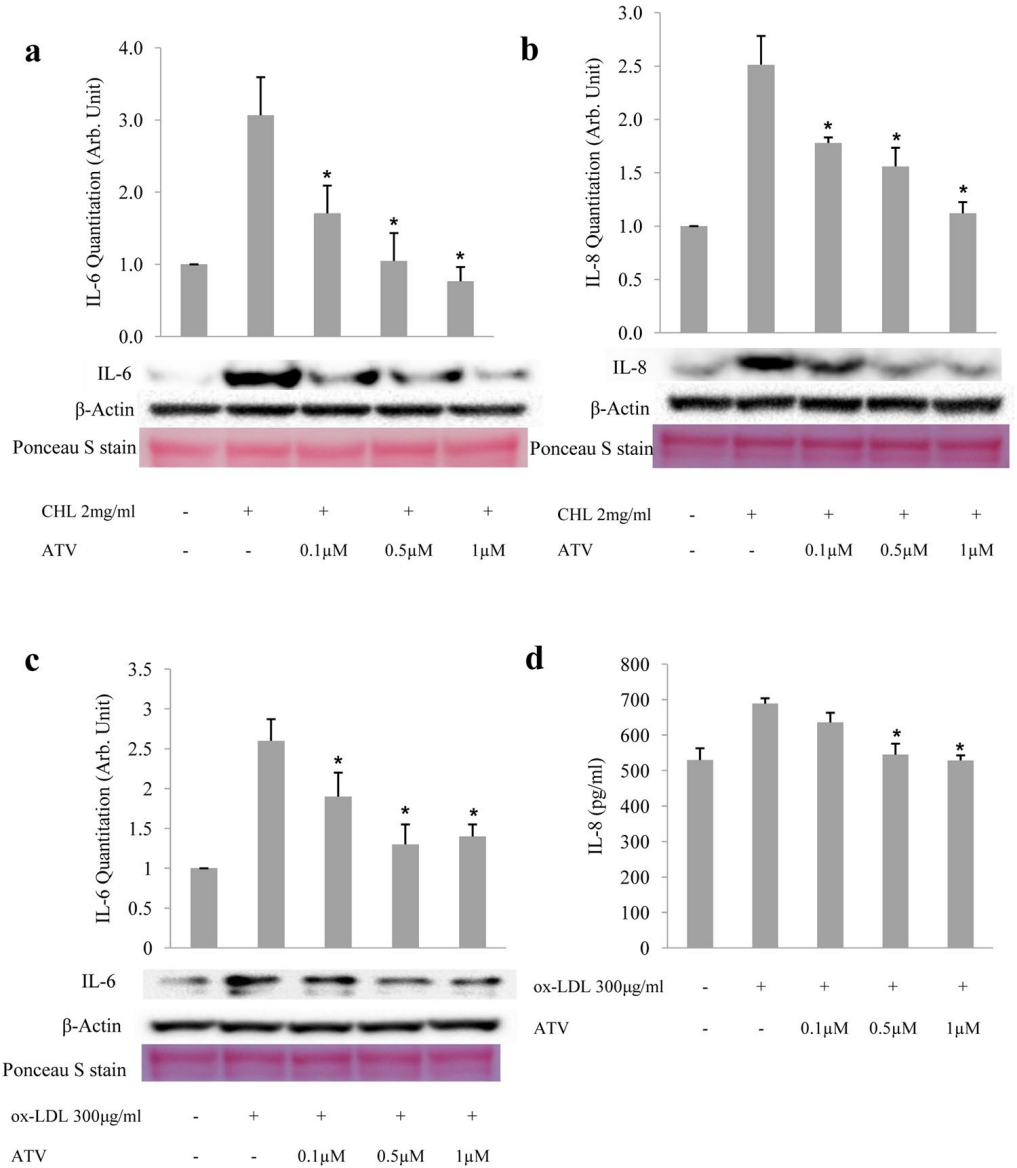
Atorvastatin inhibits IL-6 and IL-8 secretion induced by cholesterol crystals and ox-LDL in ARPE-19 cells. IL-6 (**a**) and IL-8 (**b**) secretion from ARPE-19 cells as determined by western blot of equal volumes of culture medium 6 hours after treatment with 2 mg/ml of cholesterol crystals (CHL), with or without pretreatment with 0.1, 0.5, or 1 μM of atorvastatin for 6 hours. IL-6 (**c**) and IL-8 (**d**) secretion from ARPE-19 cells, as determined by western blot and ELISA, respectively, of equal volumes of culture medium 18 hours after treatment with 300 μg/ml of ox-LDL, with or without pretreatment with 0.1, 0.5, or 1 μM of atorvastatin for 6 hours. β-actin was detected in each cell lysate. The levels of IL-6 and IL-8 are calculated after normalization against the intensity of β-actin bands. Ponceau S staining was conducted to confirm equal loading. ELISA data were normalized against the cell number. *p < 0.05 versus only CHL or ox-LDL treated group. The results are expressed as mean ± SE.

We have previously shown that cholesterol crystals induce the secretion of the inflammatory cytokines IL-6 and IL-8 in ARPE-19 cells^24^. Since these cytokines are associated with the development and progression of AMD^17, 19–23^, we investigated whether atorvastatin can block this effect. ARPE-19 cells were primed with IL-1α, treated with different concentrations of atorvastatin, and then incubated with cholesterol crystals. Τhe levels of IL-6 and IL-8 were evaluated using western blot or ELISA. As expected, cholesterol crystals increased ARPE-19 secretion of IL-6 and IL-8, 3.2- and 2.5-fold, respectively, compared to control treatment (Fig. 5a and b). However, pretreating the cells with 0.1, 0.5 or 1 µM atorvastatin significantly reduced the levels of IL-6 1.7-, 1.1 and 0.8-fold, respectively, and the levels of IL-8 1.8-, 1.6- and 1.1-fold, respectively, compared to control (*p* < 0.05) (Fig. 5a and b).

Since ox-LDL could be internalized by RPE cells and interfere with the normal cell function^13, 14^, we further investigated the effect of ox-LDL on IL-6 and IL-8 in ARPE-19 cells and the effect of atorvastatin on the resulting consequences. Similar to cholesterol crystals, incubation of ARPE-19 with ox-LDL led to a 2.6-fold increase in secreted IL-6 and promoted IL-8 from 530 to 689 pg/ml in the cell culture medium, while pretreating the cells with 0.1, 0.5 or 1 µM atorvastatin was able to reduce the levels of IL-6 1.9-, 1.3- and 1.4- fold, respectively, and of IL-8 to 636, 545 and 529 pg/ml, respectively, compared to control (*p* < 0.05) (Fig. 5c and d). Both results indicate that atorvastatin can reduce, in a dose-dependent manner, the secretion of IL-6 and IL-8 induced by cholesterol crystals and ox-LDL in ARPE-19 cells.

Taken together, these results highly suggest that atorvastatin has an anti-inflammatory role in human RPE cells challenged with inflammatory inducers.

## Discussion

Our study provides evidence that lipophilic statins enhance the phagocytic function of ARPE-19 cells, and that atorvastatin can protect these cells from the impairment of phagocytic function and the inflammatory effects induced by cholesterol crystals and ox-LDL. In addition, atorvastatin increases the membrane fluidity of ARPE-19 cells, which has been previously shown to provide an important mechanistic basis for the phagocytic capacity of scavenger cells, such as macrophages^38^.

Although RPE cells are not professional phagocytes (unlike macrophages and microglia), they are some of the most active phagocytes in the body^6, 44^. Phagocytosis of shed photoreceptor outer segments (POSs) by RPE is critical for the visual function because it preserves the retinal homeostasis, through removal of subretinal debris, namely daily shed old distal tips of POSs^6, 45, 46^. Impairment of the RPE phagocytic function results in accumulation of non-phagocytized POSs, and can cause retinal degeneration and blindness in humans as well as in animal models of retinal degeneration^6, 47–49^. Phagocytosis is also one of the most important RPE functions that are impaired in AMD^50^, and some researchers believe that drusen, the characteristic sub-RPE yellowish deposits found in AMD patients, are non-phagocytized debris that translocate from the apical surface of the RPE to the sub-RPE region of the retina^51^.

Multiple factors can negatively affect the RPE phagocytic function and contribute to the pathogenesis of AMD. Aging itself has a substantial effect on RPE phagocytosis. In an animal study for example, the phagocytic function of the RPE was found to be reduced by about 80% in aged rats compared to young rats^52^. Abnormal cholesterol homeostasis is another factor that can negatively affect the health of the RPE and is implicated in AMD^53^. Age-related accumulation of esterified and unesterified cholesterol in Bruch’s membrane, the neuroretina and choriocapillaris, especially under the macula, can lead to formation of oxysterols^54, 55^, which can also be formed during LDL oxidation^56^. Oxysterol or oxidized LDL, if internalized, can in turn directly impair the RPE function, because the progression of phagocytosis can modulate the inflammatory response as shown in “professional phagocytes”^40, 57, 58^. Furthermore, several studies have also documented that oxidized lipids as well as their metabolites have deleterious effects^54, 55, 59^. For instance, ox-LDL causes a reduction in the processing of POSs in polarized rat RPE-J cells by perturbing the fusion of lysosomes with phagosomes^15^. In our study, we showed that cholesterol crystals and ox-LDL also impede the phagocytic function of ARPE-19 cells confirming that these products negatively regulate RPE phagocytosis.

AMD is a multifactorial disease, that some investigators compare to and contrast to atherosclerosis and cardiovascular disease due to some shared similarities in risk profiles and pathobiology^60^. For this reason, the role of statins in AMD progression have been studied in multiple epidemiological and clinical studies^25–29^. However, the results are inconsistent and the evidence regarding the ability of statins to prevent or delay the onset or progression of AMD is insufficient. This could be due to multiple factors such as the heterogeneity of the disease, the lack of standardization in dosage, and/or lipophilicity of the statins. More recently, our pilot Phase1/2 clinical study showed that high-dose atorvastatin may result in resolution of drusenoid pigment epithelial detachments (PEDs) and visual improvement in a subset of high-risk subgroup of AMD patients^30^. However, this study needs verification from a larger control trial. In addition, the mechanism by which statins may function as a potential treatment in AMD is not completely understood. Atorvastatin has been shown to have a pleiotropic role regulating multiple behaviors of human RPE cells; specifically, it was found to suppress cell proliferation, adhesion, migration, and contraction^61^. Atorvastatin and simvastatin have been reported to reduce oxidative stress-induced injury to the RPE, a factor implicated in the pathogenesis of AMD, and increase RPE viability^62, 63^. Chronic administration of simvastatin to a high-fat atherogenic mouse, which develops a thickened Bruch’s membrane, improved the retinal function and ultrastructure of Bruch’s membrane, the RPE and photoreceptors^64^. Moreover, atorvastatin effectively inhibited laser-induced choroidal neovascularization (CNV), reduced macrophage infiltration into the RPE/choroid complex, and down-regulated inflammatory chemokine CCL2/MCP-1 and VEGF in mice^65^, suggesting that statins may also have a beneficial role in preventing the conversion of dry to wet AMD.

Statins are classified into lipophilic and hydrophilic. Atorvastatin, lovastatin and simvastatin are relatively lipophilic compounds, while pravastatin and rosuvastatin are more hydrophilic as a result of a polar hydroxyl group and a methane sulphonamide group, respectively^66^. This classification is important because statins of different lipophilicity can have different effects. For instance, Salman *et al*. reported that lipophilic, but not hydrophilic, statins enhance the phagocytosis function and decrease the apoptosis of human peripheral blood cells *in vitro* ^35^. In addition, lipophilic statins (like atorvastatin) are more effective than hydrophilic ones (such as pravastatin) in reducing ApoB100 secretion and cholesterol levels in cultured human RPE cells through modulation of RPE cholesterol levels^67^. Consistently, our data showed that atorvastatin, lovastatin and simvastatin all enhanced the phagocytic function of ARPE-19 cells, with simvastatin showing the strongest effect. On the other hand, the same dose of pravastatin, a hydrophilic statin, showed no significant effect on the phagocytic function of ARPE-19 cells (Fig. 1). Taken together, these data indicate that all statins are not to be considered equivalent and that choosing the appropriate statin and dose may be essential to achieving the desired effects.

Statins have been shown to affect the fluidity of cell membranes^39^ and enhance the phagocytic activity of macrophages^35, 37, 38^. Consistent with these findings, our study showed that treating ARPE-19 with 50 µM of atorvastatin increases the cell membrane fluidity significantly, thus proposing it as an important mechanism for the observed increase in the phagocytic function of ARPE-19 cells after statin treatment. Interestingly, atorvastatin did not only increase the baseline phagocytic function of ARPE-19 cells, but also preserved the phagocytic function impaired by cholesterol crystals and ox-LDL.

Although the exact pathophysiology of AMD is not known, several studies support the notion that inflammation plays an important role in the development and progression of the disease. Specifically, genetic and clinical studies have emphasized the importance of pleiotropic inflammatory cytokines IL-6 and IL-8 in the pathogenesis of AMD. In addition to its known role in inducing inflammatory and immune responses, the levels of IL-6 have been correlated with the onset^19^ and progression^17^ of AMD. IL-8 haplotypes are associated with increased risk of AMD, and the IL-8 promoter polymorphism −251A/T is an important risk factor for the disease^21^. Additionally, increased levels of IL-8 have been reported to be associated with a higher risk for developing early AMD. Moreover, IL-6 and IL-8 have been associated with wet (neo-vascular) AMD. In particular, IL-6 receptor-mediated activation of STAT3 inflammatory pathway has been found to play a significant role in the generation of CNV^68^, while intraocular concentrations of IL-6 and IL-8 (particularly IL-6) were significantly associated with the volume of macular edema in patients with CNV^69^. Furthermore, these two cytokines induce the secretion of VEGF, a known essential factor in the development of CNV^3^.

Our previous work has shown that cholesterol crystals increase IL-6 and IL-8 secretion by activating the NF-κB pathway in ARPE-19 cells^24^. In our current study, we observed that in addition to cholesterol crystals, ox-LDL can also induce IL-6 and IL-8 secretion in ARPE-19 cells. Importantly, our results showed that atorvastatin was able to decrease IL-6 and IL-8 secretion induced by cholesterol crystals and ox-LDL in ARPE-19 cells. This is consistent with clinical studies that showed that statins have anti-inflammatory properties. Specifically, Ascer *et al*. reported that atorvastatin reduces pro-inflammatory markers, such as tumor necrosis factor-alpha (TNF-α), interleukins (IL-1 and IL-6), soluble intercellular adhesion molecule-1 (sICAM-1) and C-reactive protein (CRP) in hypercholesterolemic patients^70^. Collectively, our results suggest that statins can effectively block part of the inflammatory component of AMD in RPE cells. In addition to their protective effects on the RPE phagocytic function, the anti-inflammatory properties of statins render them beneficial drugs for preserving the health and proper function of RPE cells.

In summary, this study shows that atorvastatin enhances the phagocytic function of ARPE-19 cells and is effective in protecting ARPE-19 cells against the phagocytic impairment and the inflammatory effects induced by cholesterol crystals and ox-LDL. In addition, our study suggests that the increase in cell membrane fluidity is part of the mechanism for the observed effects of statins on the phagocytic function of ARPE-19. Our results support further studies on the potential role of statins in the treatment of AMD.

## Methods

### Cell lines and reagents

The human RPE cell line ARPE-19 was purchased from ATCC (Cat#CRL-2302, Manassas, VA, US). DMEM/F-12 HEPES medium (Cat# 11330–057), fetal bovine serum (FBS; Cat# 10438–026) and penicillin-streptomycin (100 U/mL-100 μg/ml; Cat# 15140) were obtained from Life Technologies (Grand Island, NY, US). Recombinant human IL-1α was obtained from R&D Systems (Cat# 200-LA-010, Minneapolis, MN, US). Cholesterol crystals were purchased from Sigma-Aldrich (Cat# C8667, St. Louis, MO, US). Anti-IL-6 (Cat# 12153) and anti-IL-8 (Cat#4970) antibody was obtained from Cell Signaling Technology (Danvers, MA, US). Anti-IL-8 antibody (Cat# MAB208) was purchased from R&D Systems (Minneapolis, MN, US). Anti-rabbit (Cat# 7074) and anti-mouse (Cat# 7076), HRP-linked secondary antibodies were obtained from Cell Signaling Technology (Danvers, MA, US).

### Statins

Atorvastatin (Atorvastatin calcium salt trihydrate; Cat# PZ0001), simvastatin (Cat# S6196), lovastatin (Mevinolin from *Aspergillus sp*.; Cat# M2147) and pravastatin (Cat# P4498) were purchased from Sigma-Aldrich (St.Louis, MO, US). Atorvastatin, simvastatin and lovastatin were reconstituted in dimethyl sulphoxide (DMSO; Cat# 4-X, ATCC, Manassas, VA, US). Pravastatin was reconstituted in deionized water. A stock solution of 5 mM of each statin was prepared, and further dilutions were made in medium. Culture medium in the control group were supplemented with either DMSO or deionized water consistent with the solvent utilized to dissolve the statins, and at final concentrations corresponding to the highest concentrations added to the statins.

### Cell culture

ARPE-19 cells were maintained in DMEM/F-12, HEPES medium supplemented with 10% FBS, 100-U/mL penicillin and 100-μg/mL streptomycin. The cells were grown in humidified 5% CO_2_ at 37 °C, and passaged when reaching 80% confluence.

### Preparation of cholesterol crystals solution

Cholesterol crystals were pulverized finely with a grinder and subsequently sterilized with UV light for 30 minutes. ARPE-19 culture medium was added to the cholesterol crystals to make a 2–6 mg/mL stock solution. The stock solution was sonicated directly with a sterile probe (Model# CL334, Qsonica, Newtown, CT) connected to Q500 Sonicator (Model # Q500–110, Qsonica, Newtown, CT) with the amplitude set at 25%, until the cholesterol crystals were evenly suspended in the culture medium.

### Preparation of oxidized LDLs

LDLs (Cat# 360–10, LEE Biosolutions, Maryland Heights, MO, US) were oxidized using CuSO_4_ (Cat# 1297, Sigma-Aldrich, St. Louis, MO, US), as previously described^71^. Briefly, LDL (600 µl, 0.25 mg/ml), CuSO_4_ (22.5 µl, 1.6 mM) and DPBS (277.5 µl) were mixed and incubated at 37 °C. The oxidation reaction was stopped using 1 mM of EDTA after 24 hours of incubation. Immediately after oxidation, lipoproteins were desalted using PD-10 disposable desalting columns (Cat# 17-0851-01, GE Healthcare, Buckinghamshire, UK).

### Confocal microscopy

ARPE-19 cells were seeded in a 4-well chamber slide (Cat#PEZGS0496, EMD Millipore, Billerica, MA) and cultured for 24 hours to 80–90% confluence. Cells were subsequently incubated with 5 × 10^7^/ml of 1 µm-diameter Fluoresbrite® YG Carboxylate Microspheres (Cat#15702, Polysciences, Warrington, PA, US) for 6 hours. The cells were then washed with DPBS (Cat# BE17-515Q, Lonza, Walkersville, MD, US) three times, after which 4% paraformaldehyde (Cat# 28906, Thermo Fisher Scientific, Rockford, IL, US) was used to fix the cells for five minutes. Then, the cells were washed with DPBS 3 times (5 minutes for each wash). Finally, cells were stained with DAPI (diamidino-2-phenylindole, Cat# 62248, Thermo Fisher Scientific, Germany) for 2 minutes. After a brief rinse with DPBS, cells were mounted under a coverslip with Vectashield mounting medium (Cat# H-1000, Vector Laboratories, Burlingame, CA, US). The cell slide was observed and imaged using Leica TCS SP5 laser-scanning confocal microscope (Leica, Wetzlar, Germany). A Z-stack of 50 optical sections was collected from the bottom of the culture chamber toward the top of the slide to include the entire cell layer. The Leica Application Suite (Version 2.6.0) software was used to collect the images. The final images were assembled with Image J (Version 2.0.0).

### Phagocytosis analysis by flow cytometry

A flow cytometric assay was used to evaluate cell phagocytosis according to a protocol described by Mukherjee *et al*.^46^. Briefly, ARPE-19 cells were seeded in 12-well plates and cultured until 90% confluent. Cells were incubated for 6 hours with 5 × 10^7^/ml of 1 µm-diameter Fluoresbrite® YG Carboxylate Microspheres (Cat#15702, Polysciences, Warrington, PA, US) alone, or in combination with 50 µM of different statins (atorvastatin, lovastatin, simvastatin or pravastatin). For the dose-dependent effect of atorvastatin on ARPE-19 phagocytic function, the same experimental procedure was followed, but using various concentrations (1, 25, 50, or 75 µM) of atorvastatin.

To study the effect of cholesterol crystals or ox-LDL on the phagocytic function of ARPE-19 and the protective effect of atorvastatin against these stimuli, cells were treated with 50 µM atorvastatin or vehicle control for 6 hours. Next, they were treated with 2 mg/ml cholesterol crystals and incubated with the Fluoresbrite® YG Carboxylate Microspheres for 6 hours, or treated with 300 µg/ml ox-LDL for 18 hours and incubated with the Carboxylate Microsphere during the last 6 hours of ox-LDL treatment. In all previous experiments after incubation, cells were washed with PBS three times to remove any extracellular beads, then trypsinized (0.25% trypsin-EDTA; Cat# 25200–056, Gibco, Grand Island, NY, US) for 1 minute, and neutralized with pre-warmed culture medium. The cell suspension was collected and centrifuged (241 g, 5 mins). The cell pellet was re-suspended in 0.5 ml PBS for each sample. Subsequently, the cells were analyzed for green phagocytized fluorescent beads (excitation wavelength of 441 nm and emission wavelength of 486 nm) by a FACScalibur flow cytometer using the CellQuest 3.0.1(Becton & Dickinson, Mountain View, CA, US) and FlowJo 10.0 software. The percent of phagocytic cells present in each group was recorded as well as the mean fluorescence intensity of engulfed particles.

### Fluorescence Recovery After Photo-bleaching (FRAP) to assess cell membrane fluidity

ARPE-19 cells were cultured onto 35 mm glass bottom dishes (Cat# P35G-1.5-14-C, MatTek Corporation, Ashland, MA, US) in DMEM/F12 medium supplemented with 10% FBS and 1% penicillin/ streptomycin. 70–80% confluent cells were treated with 50 μM atorvastatin for 3 hours. Thirty minutes before measuring the membrane fluidity, cells were incubated with 5 μM of 4, 4-difluoro-5, 7-dimethyl-4-bora-3a, 4a-diazas-indacene-3-dodecanoic acid (BODIPY® FL C12) (Cat#D3822, Thermo Fisher Scientific, Waltham, MA), a green fluorophore combined with a 12-carbon saturated hydrocarbon tail, dissolved in FluoroBrite^TM^ DMEM medium (Cat#A1896701, Thermo Fisher Scientific, Waltham, MA). Then, cells were washed with FluoroBrite^TM^ DMEM medium to remove any unincorporated dye, and were maintained at 37 °C and 5% CO_2_ using an environmental chamber mounted on a microscope stage for FRAP measurements. FRAP measurements were made by photo-bleaching a microscopic area of the cell membrane [bleach Regions of Interest (ROIs)] were drawn using a 7 × 7 pixel square) with a short (2 seconds), intensive pulse of light (458, 477, 488, and 514 nm lines simultaneously at 100% transmission) from an argon laser through a 63×, 1.4 numerical aperture oil immersion objective -with an additional 2× digital zoom (Total magnification = [10×][63×][2×] = 1,260×) of a Zeiss LSM 510 Axiovert 200 M confocal laser scanning microscope. Recovery of fluorescence within the bleached area, due to lateral diffusion of neighboring intact fluorophore, was assessed by repetitive scanning across the cell surface (every 500 ms) with an attenuated laser beam (488 nm line at 3% transmission).

### Western blot

ARPE-19 cells were seeded in 6-well plates at a density of 1 × 10^5^ cells/well and equal volumes of cell culture medium per well were used throughout the experiment. Twenty-four hours later, the cells were primed with IL-1α (5 ng/ml) for 8 hours, treated with atorvastatin (0.1, 0.5, or 5 μM) or vehicle control for 16 hours, and then incubated with either 2 mg/ml of cholesterol crystals for 6 hours, or 300 µg/ml oxLDL for 18 hours, with or without atorvastatin. After treatment, the culture medium was collected and centrifuged at 13.3 g for 15 minutes at 4 °C. After the culture medium was collected, equal volume of NP40 cell lysis buffer (Cat#FNN0021, Invitrogen, Frederick, MD) was added to each culture well, and cell lysates were collected to detect β-actin for normalization. Equal volumes of culture medium (for IL-6, IL-8) or extracted protein (for β-actin) were loaded on each lane and the samples were run by electrophoresis. The proteins were transferred to a PVDF membrane. Ponceau S staining of the PVDF membrane was performed to check for equal loading of protein from culture medium transfer. Subsequently, the membrane was blocked with non-fat milk (Cat#9999, Cell signaling technology, Danvers, MA) and incubated with primary antibodies against IL-6, IL-8 or β-actin. The membrane was then washed and incubated with HRP-conjugated secondary antibodies at room temperature for 60 minutes. The membrane was developed with enhanced chemiluminescence (Cat#RPN2232, ECL Prime western blotting detection reagents, GE Healthcare Life Sciences, Piscataway, NJ). The intensity of protein bands was measured using the software Image Lab 4.1 (Bio-Rad, Hercules, CA, US).

### Enzyme-linked Immunosorbent Assay (ELISA)

IL-8 secretion induced by ox-LDL was measured by analysis of conditioned media of ARPE-19 cells using an ELISA kit (Cat# D8000C, R&D Systems, Minneapolis, MN, US) according to the manufacturer’s instructions.

### Statistical Analysis

All experiments were performed in triplicate. Statistical analyses were performed using GraphPad Prism 5.0a. The results are expressed as mean ± SE. The statistically significant difference between two-treatment groups was analyzed by unpaired *t* test. The value of *p* < 0.05 was set as statistically significant.
